# NAP1L5 targeting combined with MYH9 Inhibit HCC progression through PI3K/AKT/mTOR signaling pathway

**DOI:** 10.18632/aging.204377

**Published:** 2022-11-11

**Authors:** Rui Zhao, Yuzhen Ge, Yongjun Gong, Bo Li, Benli Xiao, Shi Zuo

**Affiliations:** 1 Guizhou Medical University, Guiyang, Guizhou 550001, China; 2 Department of Hepatobiliary Surgery, The Affiliated Hospital of Guizhou Medical University, Guiyang, Guizhou 550001, China

**Keywords:** hepatocellular carcinoma, Nap1L5, MYH9, proliferation, migration, invasion

## Abstract

Hepatocellular carcinoma (HCC) is one of the leading causes of cancer death worldwide. Nucleosome assembly protein 1-like 5 (NAP1L5) is a protein-coding gene that encodes a protein similar to nucleosome assembly protein 1 (NAP1). It is a histone chaperone that plays an important role in gene transcription in organisms. However, the role of NAP1L5 in the pathogenesis of hepatocellular carcinoma remains to be elucidated. In this study, low expression of NAP1L5 was found in hepatocellular carcinoma, and the downregulation of NAP1L5 was related to shorter survival and disease-free survival. In addition, its expression is also related to the tumor size and recurrence of hepatocellular carcinoma. The overexpression and knockdown of NAP1L5 by plasmid and siRNA showed that NAP1L5 inhibited the proliferation, migration and invasion and induced apoptosis of hepatoma cells. *In vivo* experiments confirmed that NAP1L5 can inhibit the growth and metastasis of hepatocellular carcinoma cells. In the mechanistic study, we found that NAP1L5 affects the occurrence and development of hepatocellular carcinoma by regulating MYH9 to inhibit the PI3K/AKT/mTOR signaling pathway. As a functional tumor suppressor, NAP1L5 is expressed at low levels in HCC. NAP1L5 inhibits the PI3K/AKT/mTOR signaling pathway in hepatocellular carcinoma by regulating MYH9. It may be a new potential target for liver cancer treatment.

## INTRODUCTION

According to recent statistics, there are approximately 906000 new cases of liver cancer and 830000 related deaths every year; thus, liver cancer has become a serious threat to human health [1]. Although the comprehensive treatment of liver cancer has made great progress, the prognosis of liver cancer is still not ideal. Therefore, understanding the molecular mechanism and changes in the occurrence and development of HCC is very important to improve the treatment and prognosis of patients with liver cancer.

Nucleosome assembly protein 1 (NAP1) is recognized as a histone chaperone. It has been found that histone chaperones are proteins that bind and transport histones, preventing the chaotic aggregation of histones during nucleosome formation [2]. It contains a highly conserved central NAP domain, which is an important binding site necessary for histone binding and nucleosome assembly and plays a key role in maintaining gene transcription in eukaryotes [3]. Recent studies have demonstrated at least five homologous Nap-1 genes in mice and humans [4], namely, NAP1L1, NAP1L2, NAP1L3, NAP1L4 and NAP1L5. After studying their specific functions, we found that the mouse NAP1L2 protein can bind to chromatin in S phase during chromatin condensation, regulate neuronal cell proliferation and play an important role in neural tube development and neuronal differentiation [5]. The mouse NAP1L5 gene was identified as a paternally imprinted gene in mouse parthenogenetic embryos for the first time [6]. With further study of the NAP1 family in humans, it was found that PRDM8 exhibited antitumor activity in HCC by regulating NAP1L1 [7]. LncRNA CDKN2B-AS1 promotes proliferation and metastasis of human hepatocellular carcinoma through the let-7c-5p/NAP1L1 axis [8]. NAP1L1 and NAP1L4 regulate apoptosis by selectively regulating the expression of the p53 pre-response block and proapoptotic genes in normal homeostasis and stress-induced responses [9]. NAP1L4 acts as a histone chaperone throughout the cell cycle, and its expression level increases with the progression of the cell cycle from G0/G1 to M phase [10]. Currently, there are few studies on human NAP1L5. The human Nap1l5 gene is a patrilineal imprinted gene located in 4q22.1 [6]. This region is the target of intermediate gene loss in malignant liver tumors, such as hepatocellular carcinoma and hepatoblastoma, suggesting that there may be suppressor genes for liver cancer [11]. To date, the role and mechanism of human NAP1L5 in the progression of hepatocellular carcinoma are not clear.

In this study, we found that the expression level of NAP1L5 in HCC tissues was lower than that in normal tissues, and the expression of NAP1L5 was closely related to the prognosis of HCC. NAP1L5 inhibits the malignant biological characteristics of hepatocellular carcinoma cells *in vivo* and *in vitro*. In addition, NAP1L5 induced a decrease in cells in the S phase of the cell cycle and promoted the apoptosis of HCC cells. According to the mechanistic analysis, NAP1L5 suppresses the PI3K/AKT/mTOR signaling pathway through targeted regulation of MYH9. Our findings suggest that NAP1L5 may become a potential biomarker for liver cancer diagnosis and a new target for treatment in the future.

## MATERIALS AND METHODS

### Patients and specimens

The tissues of hepatocellular carcinoma and paracancerous liver tissues of patients treated in the affiliated Hospital of Guizhou Medical University in 2021 were collected and assessed by Western blotting (*n* = 8). The collected samples were stored at −80°C for further study. A tissue microarray from Shanghai Outdo Biotech Co., Ltd. (China), was used to analyze a total of 90 pairs of primary liver cancer tissues and the related clinical and prognostic data.

### Cell lines and cell culture

LO2 normal human hepatocytes and HuH7, PLC/PRF5, MHCC97H, HLF, LM3, and HepG2 hepatoma cells were purchased from Zhejiang Meisen Cell Technology Co., Ltd. (Zhejiang, China). We used high-glucose Dulbecco’s modified Eagle medium containing 10% fetal bovine serum and 1% streptomycin solution/Corning penicillin to culture the cells. The cells were cultured in a humidified chamber with a concentration of 5% carbon dioxide at 37°C.

### Lentivirus transduction, plasmids and small interfering RNA (siRNA) transfection

To construct a stable overexpression cell line, lentivirus was used as a vector to carry the NAP1L5 sequence, and empty vector was used as a negative control. The full-length CDS of Flag-NAP1L5 with green fluorescent protein (GFP) was inserted into a lentiviral vector (GENE, Shanghai, China). The lentiviral vector was introduced into HepG2 and MHCC97H cells at MOI of 10, and the original culture medium was replaced with fresh culture medium 24 hours later. The plasmid was purchased from Designgene Biological Co., Ltd. (Wuhan, China), and siRNA was synthesized by RiboBio (Guangzhou, China). According to the operating procedures provided in the manufacturer’s instructions, the plasmid and siRNA were introduced into HepG2 and MHCC97H cells with liposome 3000 reagent (Invitrogen). The siNAP1L5, siMYH9 and control sequences were recombined into HepG2 and MHCC97H cells, and the sequences were as follows:

st-h-NAP1L5_001: AGCCAAATTTGATAAGGAA
st-h-NAP1L5_002: GAAGCGATGCGATAAGATA
st-h-NAP1L5_003: GCCTGCCTAATTCGGTGAA
st-h-MYH9: CGCGTCCCCGCAAACCTCGAGAAGGCAATTCAAGAGATTGCCTTCTCGAGGTTTGCTTTTTGGAAAT

### Western blotting

According to the instructions, phosphorylated and unphosphorylated protease inhibitors were added to RIPA protein lysate to extract total protein. The working solution of BCA was prepared, and the protein concentration was determined by a BCA protein concentration detection kit (Boster, Wuhan, China). The protein concentration was determined by the BCA method, and the absorbance of other wavelengths between 540 and 595 nm was determined by an enzyme-labeling instrument. The protein concentration of the sample was calculated according to the standard curve, sample volume and dilution multiple. A sodium dodecyl sulfate-polyacrylamide SDS–PAGE gel was prepared, and the protein was then separated by electrophoresis (80 V, 120 min) and transferred to a polyvinylidene fluoride (PVDF) membrane (Bedford Millikon, MA, USA). After cutting, the membranes were rinsed with TBST, 5% skim milk powder was added, and the membranes were sealed for 2 hours. After TBST cleaning, the primary antibody was added and incubated overnight at 4°C. TBST was cleaned after the secondary antibody was added and incubated at room temperature for 2 h. An (ECL)-Plus kit (Abclonal, Wuhan, China) was used for fluorescence detection. Primary antibodies against the following proteins were used:

NAP1L5 (1:1000, Abclonal, A17849), GAPDH (1:50000, Abclonal, AC033), AKT (1:1000, Proteintech, 10176-2-AP), Phospho-akt (1:2000, Proteintech, 66444-1-Ig), mTor(1:2000, Proteintech, 28273-1-AP), Phospho-mTOR (1:2000, Proteintech, 80596-1-RR), CDK4 (1:1000, Proteintech, 11026-1-AP), CDK6 (1:1000, Proteintech, 14052-1-AP), CyclinD1 (1:1000, Proteintech, 26939-1-AP), N-Cadherin (1:2000, Proteintech, 22018-1-AP), E-Cadherin (1:5000, Proteintech, 20874-1-AP), Vimentin (1:2000, Proteintech, 10366-1-AP), SNAIL1 (1:1000, Proteintech, 26183-1-AP), BAX (1:1000, Proteintech, 50599-2-Ig), BCL2 (1:1000, Proteintech, 12789-1-AP), and β-tublin (1:3000, Proteintech, 10094-1-AP).

### Immunohistochemistry

The expression of NAP1L5, Ki67 and TUNEL was detected by immunohistochemistry (IHC). Carcinoma and tumor transplantation tissues were fixed with paraformaldehyde, embedded in paraffin, and sliced at 4 microns for immunohistochemical detection. After dewaxing, hydration, and antigen repair, peroxidase-labeled streptavidin was added, and the sections were blocked with goat serum. Tissue sections were incubated with primary antibodies (NAP1L5 (1:200), Ki67 (1:1200) and TUNEL (1:200)) in a refrigerator at 4°C for one night, incubated with secondary antibodies at room temperature for 20 min, and then incubated with chromogenic diaminodiamine for 5 min to detect antigens.

### RT-qPCR

Using TRIzol reagent (Vazyme, Nanjing, China), total RNA was extracted from liver cancer cell lines according to the manufacturer’s instructions. RNA was reverse transcribed into cDNA by superscript III RT (Vazyme, Nanjing, China) and random primers. Then, quantitative PCR with SYBR PreMix Ex Taq (Vazyme, Nanjing, China) was carried out by using an iQ5 quantitative RT–qPCR detection system (Bio-Rad, Richmond, CA, USA). The relative expression of genes was analyzed by the 2^−ΔΔCT^ method. The primer sequence was as follows: NAP1L5 forward primer ATGGCCGACTCGGAAAACC; reverse primer TAGGCAGGCTCTCGATAAAGT; GAPDH forward primer GGAGCGAGATCCCTCCAAAAT; reverse primer GGCTGTTGTCATACTTCTCATGG.

### Colony formation assay

The cells were inoculated in a 6-well culture plate with 500 cells in each well. After incubation in high-glucose DMEM for 14 days, the cells were washed with phosphate-buffered saline (PBS) 3 times, fixed with 4% paraformaldehyde and stained with hematoxylin. The number of communities was counted under a light microscope.

### Migration and invasion assays

In the wound healing migration assay, approximately 1 × 106 HCC cells were seeded in a six-well culture plate. After the hepatoma cells were attached, the tip of a 200-μL plastic straw was scraped along the bottom of the plate to form a wound. The cells were washed with PBS 3 times, the isolated cells were removed, and serum-free medium was added. Forty-eight hours later, the healing area was calculated. In the Transwell assay, a 24-well Transwell filter and Matrigel were used for cell migration and invasion assays. The cells (5 × 104) were inoculated in 200 μL of serum-free medium, and 700 μL of medium containing 10% fetal bovine serum was added and incubated at 37°C for 48 h (migration test) or 24 h (invasion test). The cells on the submembrane surface were stained with 0.1% crystal violet and photographed, and each group was randomly divided into 4 visual fields under a light microscope.

### EdU staining

The BeyoClick EdU cell proliferation kit was used in conjunction with Alexa Fluor 555 for EdU staining according to the manufacturer’s instructions. Briefly, 2 × 105 hepatoma cells were inoculated into a 6-well plate with cover slides. After 12 hours, 10 μM EDU chromogenic agent was added to each well, incubated at 37°C for 1.5 hours, washed with PBS, fixed with 4% paraformaldehyde, permeabilized with 0.3% Triton X-100 and stained with Click additive. The nucleus was stained for 10 min with Hoechst 33342, and EDU-positive cells were counted under a fluorescence microscope.

### Cell apoptosis assays and cell cycle

Apoptosis: A total of 2 × 104 cells were collected, and the living cell culture medium was centrifuged with the digested cell suspension, washed with PBS 3 times, and stained with 7 μl of Annexin V and 10 μl of 7-AAD for 10 min. The apoptosis rate was detected by a flow CytoFLEXS cytometer and FlowJo software. Cell cycle: A total of 106 cells were prepared, washed and resuscitated with PBS, and the cells were collected by centrifugation at 300 × g for 5 min. The cells were completely resuscitated with PBS precooled at 2 ml at 4°C and centrifuged at 300 × g for 5 minutes. The supernatant was discarded, and some PBS was resuspended to form a single-cell suspension. After the cells were fully dispersed into a single-cell suspension, they were slowly dripped into anhydrous ethanol precooled at −20°C, kept at a concentration of 70–75% PBS overnight at −20°C, washed twice with PBS, and centrifuged for 300 × g for 5 min. Then, 200–400 μL of PI/RNase staining solution was added, and the cells were resuscitated and stained for 30 minutes. Within 24 hours, the cells were detected by flow cytometry at an excitation wavelength of 488 nm and an emission wavelength of 585 ± 21 nm. The cell cycle distribution was determined by the cell cycle fitting software Kaluza and FlowJo.

### Animal experiments

In the tumor growth experiment, MHCC97H cells carrying NAP1L5 high-expression lentivirus vector or control vector were subcutaneously inoculated into the right acromion of nude mice in the experimental and control groups (4-week-old BALB/c-nude, 5 mice/group, 2.0 × 106/mouse). Tumor formation in mice was observed for more than 28 days. The tumor volume was monitored every 4 days, and the formula was as follows: V = (length × width^2^)/2 mm^3^. In the *in vivo* transfer experiment, approximately 150 μL of MHCC97H cells with a high expression of NAP1L5 or carrying control vector were injected into the tail vein of mice in the experimental and control groups (4-week-old BALB/c-nude, 5 mice/group, 2.0 × 106/mouse). After 4 weeks, the mice were killed, and lung tissue was collected to observe and count metastasis. The lung tissue was stained with HE.

### Coimmunoprecipitation (Co-IP)

The cells were collected and cleaved in low-salt lysis buffer for 30 min at 4°C. The samples were incubated with immunoglobulin microspheres at 4°C for 2 hours and then centrifuged at 5000 rpm for 10 minutes. The supernatant (20 μl) was saved as “Input”. Anti-IgG antibodies (Proteintech, Wuhan, China) or anti-Flag antibodies (Proteintech, Wuhan, China) were added to the remaining cell lysate and prewashed with TBST buffer 3 times. The cell lysate with antibody was rotated gently overnight at 4°C. The protein A/G microspheres were added to the cell lysate and then rotated at 4°C for 4 hours to harvest microbeads. The beads were washed in TBST 3 times and then boiled for 10 min in 2X sodium dodecyl sulfate loading buffer. The follow-up analysis was performed by Western blotting.

### Mass spectrometry

MHCC97H cells expressing NAP1L5 marked by FLAG were collected and lysed at 4°C. Then, according to the instructions of the manufacturer, IP experiments were carried out with a FLAGIP Kit (Sigma, FLAGIPT1). The lysate of MHCC97H cells overexpressing NAP1L5 labeled with FLAG was centrifuged at 12000 rpm for 10 min in a test tube. The supernatant was transferred to a fresh test tube and sent to Shanghai BGI Tech Solutions Co., Ltd., for mass spectrometry and further analysis.

### Statistical analysis

All the data were analyzed by GraphPad Prism 8 and SPSS version 19.0 (IBM Corp., Armonk, NY, USA). The differences between groups were compared by the double-tailed Student’s *t* test. Multiple groups were compared by single-factor analysis of variance (ANOVA). *P* values <0.05, <0.01, and <0.001 indicated statistical significance.

### Availability of data and materials

All data are available upon request.

## RESULTS

### The downregulated expression of NAP1L5 in hepatocellular carcinoma is related to poor prognosis

**Figure 1:**
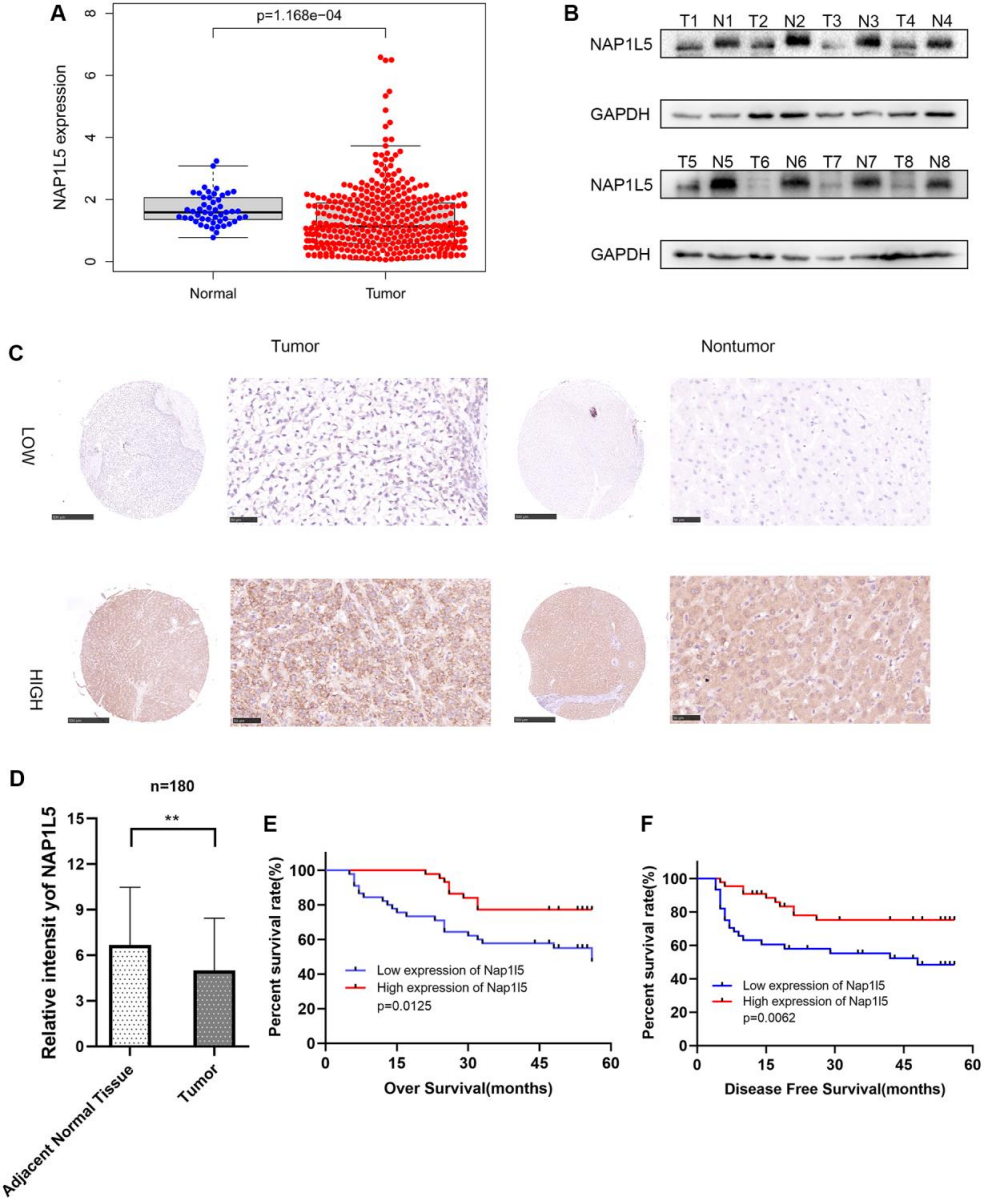
**The expression of NAP1L5 in hepatocellular carcinoma is decreased and is related to poor prognosis.** (**A**) The expression of the NAP1L5 gene in the TCGA database: 50 paracancerous tissues and 374 cancerous tissues. (**B**) The expression of NAP1L5 protein in 8 pairs of hepatocellular carcinoma (Tumor) and paracancerous tissues (Normal) was assessed. (**C**) Immunohistochemistry was used to detect the expression of NAP1L5 in the HCC tissue microarray. (**D**) The immunohistochemical score of 90 samples of HCC and paracancerous tissues in the tissue microarray. (**E**, **F**) Kaplan–Meier survival analysis was performed to evaluate the effect of NAP1L5 on disease-free survival and overall survival. ^*^p < 0.05; ^**^p < 0.01;^***^p < 0.001.

We searched the TCGA database for the RNA expression of NAP1L5 in hepatocellular carcinoma and found that the expression of NAP1L5 in cancer tissues was lower than that in adjacent tissues (Figure 1A). We collected 8 pairs of hepatocellular carcinoma tissue samples from patients with hepatocellular carcinoma and corresponding paracancerous tissues from the affiliated Hospital of Guizhou Medical University. We conducted Western blotting to detect the NAP1L5 protein. The protein content of NAP1L5 in cancer tissue was lower than that in adjacent tissue (Figure 1B). We performed immunohistochemistry in 90 pairs of hepatocellular carcinoma tissue microarrays. Figure 1C shows hepatocellular carcinoma and paracancerous tissues with low and high expression of NAP1L5 (Figure 1C). After scoring, we found that the MAP1L5 score in liver cancer tissues was significantly lower than that in adjacent tissues, with scores of 6.678 and 5.000, respectively, and the difference was statistically significant (Figure 1D). We concluded that the overall survival rate of the low-NAP1L5 group was low, and the disease-free survival rate of the low-NAP1L5 group was significantly lower than that of the high-NAP1L5 group by Kaplan–Meier survival analysis (Figure 1E, 1F). According to the immunohistochemistry results, 89 cases of hepatocellular carcinoma were divided into a low expression group (*n* = 45) and a high expression group (*n* = 44). Interestingly, the group with low expression of NAP1L5 had a lower survival rate and a higher recurrence rate (Table 1). Univariate Cox regression analysis showed that the tumor volume in the group with low expression of NAP1L5 was larger (hazard ratio [HR] 2.086, 95% confidence interval [CI] 1.017–4.276% confidence interval 0.045), and the AJCC stage was later (risk ratio [HR] 2.262 min, 95% confidence interval [CI], 1.128–4.536). The histological grade was higher (risk ratio [HR] 3.028 pcm, 95% confidence interval [CI], 1.382–6.633%, confidence interval [CI], 1.382–6.633%, confidence interval [CI] 4.860–1565.433%, confidence interval [CI] 4.860–1565.433% confidence interval) (Table 2).

**Table 1:** NAP1L5 expression with clinicopathological characteristics of patients with HCC.

**Variable**	**All cases**	**NAP1L5 density**		***p*-value**
		**Low**	**High**	
		***n* = 45**	***n* = 44**	
**Age (years)**				0.900
≦50		19	18	
>50		26	26	
**Gender**				0.276
Female		3	6	
Male		42	38	
**Vital Status**				0.018
Alive		24	34	
Dead		21	10	
**HbsAg**				0.471
Positive		11	8	
Negative		34	36	
**AFP (ng/mL)**				0.937
≦400		28	29	
>400		16	16	
**ALT level (U/L)**				0.578
≦40		25	27	
>40		20	17	
**Cirrhosis**				0.699
Yes		4	5	
No		41	39	
**Tumor size (cm)**				0.667
≤6		33	34	
>6		12	10	
**AJCC Stage**				0.691
I		31	32	
II–III		14	12	
**Histological Grade**				0.169
I–II		18	24	
III		27	20	
**Involucrum**				0.169
Complete		27	20	
Incomplete or absent		18	24	
**Recurrence**				0.015
Yes		15	26	
No		30	18	

We divided the patients into high expression group and low expression group according to the expression of NAP1L5. Patients and disease were compared in each group.

**Table 2:** Univariate analysis.

	***p***	**Hazard ratio**	**95% confidence interval**
Nap1l5	0.017^*^	0.397	0.186–0.846
Age	0.676	1.165	0.569–2.384
Gender	0.371	1.923	0.459–8.052
HbsAg	0.936	0.966	0.412–2.263
AFP (ng/mL)	0.906	1.045	0.501–2.182
ALT level (U/L)	0.349	1.401	0.692–2.835
Cirrhosis	0.439	1.762	0.420–7.388
Tumor size (cm)	0.045^*^	2.086	1.017–4.276
AJCC Stage	0.021^*^	2.262	1.128–4.536
Histological Grade	0.006^*^	3.028	1.382–6.633
Involucrum	0.066	0.500	0.239–1.046
Recurrence	0.002^*^	87.233	4.860–1565.433

Corresponding staining results were grouped according to tissue chip histochemistry score. Patients were evaluated for correlation factor analysis using grouping results.

### The inhibitory effect of NAP1L5 on cell proliferation, migration and invasion *in vitro* was observed

**Figure 2:**
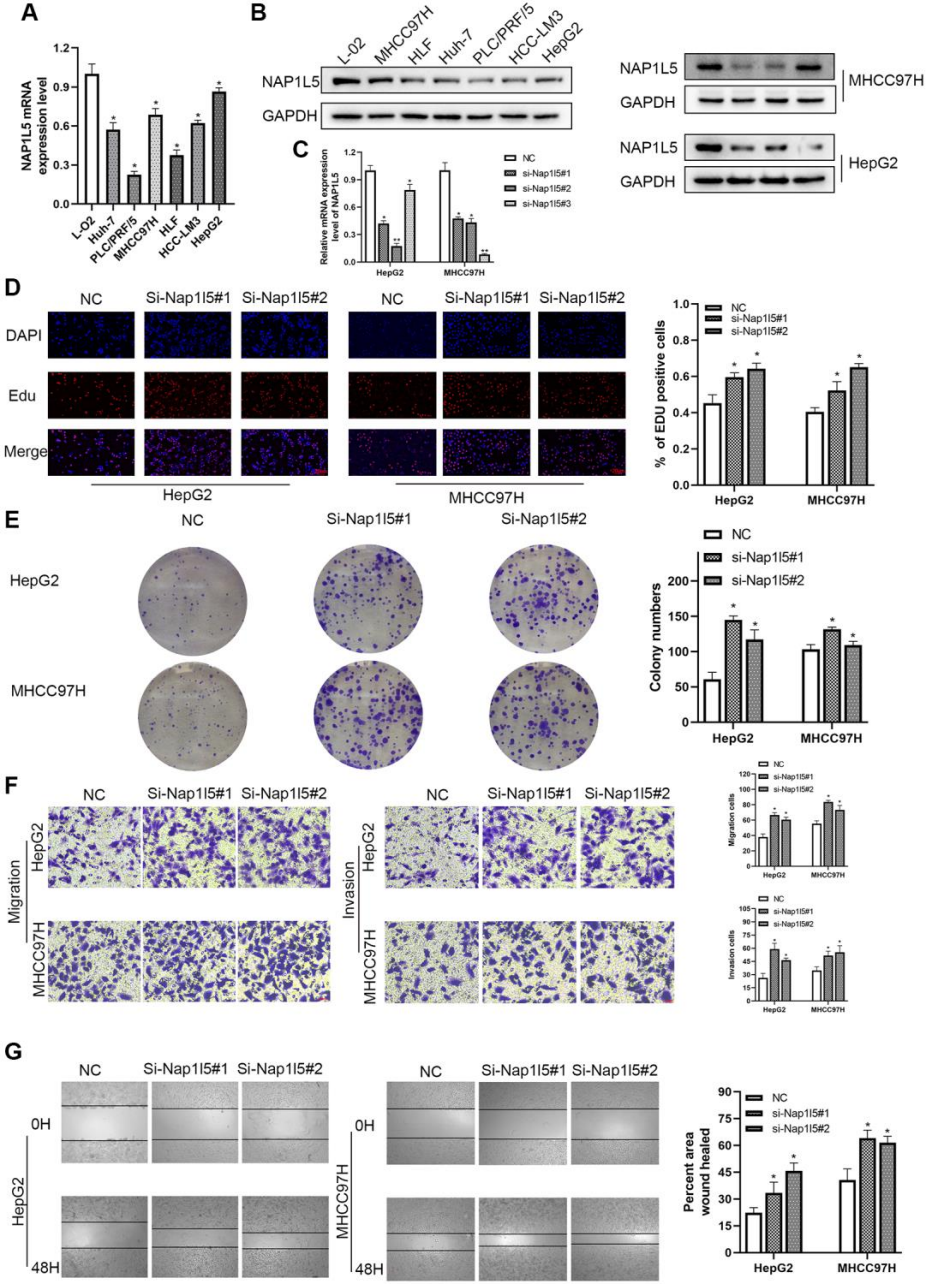
**Downregulation of NAP1L5 can promote the proliferation, migration and invasion of hepatocellular carcinoma cells *in vitro*.** (**A**) NAP1L5 mRNA expression in the LO2 and hepatoma cell lines. (**B**) The expression of NAP1L5 protein in the LO2 and hepatoma cell lines. (**C**) Validation of siRNaA transfection efficiency. (**D**) The effect of the downregulation of NAP1L5 on the proliferation of HepG2 and MHCC97H cells was detected by EdU staining. (**E**) A colony formation assay was used to detect the effect of NAP1L5 downregulation on the proliferation of HepG2 and MHCC97H cells. (**F**) Transwell assays were used to detect the effect of NAP1L5 downregulation on the migration and invasion of HepG2 and MHCC97H cells. (**G**) The effect of the downregulation of NAP1L5 on the migration of HepG2 and MHCC97H cells was detected by a wound healing migration assay. ^*^p < 0.05; ^**^p < 0.01; ^***^p < 0.001.

**Figure 3:**
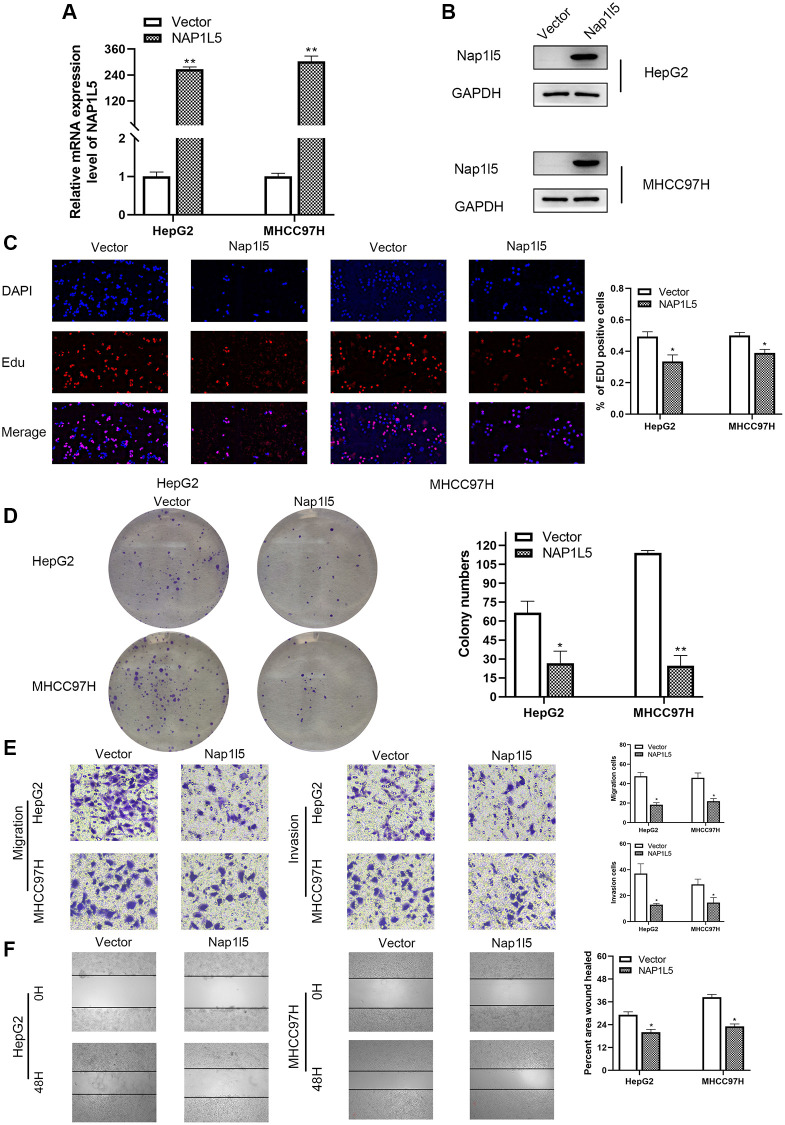
***In vitro*, NAP1L5 overexpression inhibited cell proliferation, migration and invasion.** (**A**) The mRNA expression of NAP1L5 in HepG2 and MHCC97H cell lines after plasmid transfer into vector and NAP1L5. (**B**) The expression of NAP1L5 protein in HepG2 and MHCC97H cell lines after plasmid transfer into vector and NAP1L5. (**C**) EdU staining was used to detect the effect of NAP1L5 overexpression on the proliferation of HepG2 and MHCC97H cells. (**D**) The effect of NAP1L5 overexpression on the proliferation of HepG2 and MHCC97H cells was detected by a colony formation assay. (**E**) Transwell assays were performed to detect the effect of NAP1L5 overexpression on the migration and invasion of HepG2 and MHCC97H cells. (**F**) The effects of NAP1L5 overexpression on the migration and invasion of HepG2 and MHCC97H cells were detected by a wound healing migration assay. ^*^p < 0.05; ^**^p < 0.01; ^***^p < 0.001.

We used RT–qPCR and Western blotting to study the expression of NAP1L5 in hepatocellular carcinoma cell lines and noncancerous liver cell lines. We found that the mRNA and protein expression levels of NAP1L5 in hepatocellular carcinoma cell lines were lower than those in noncancer cell lines (Figure 2A, 2B). MHCC97H and HepG2 cells were selected, and lentivirus carrying small interfering RNA (siRNA) was used to knock down the expression level of NAP1L5. Compared with the negative control at the mRNA and protein levels, the knockdown efficiency of three NAP1L5 siRNAs was confirmed (Figure 2C). Among the three siRNAs tested, siRNA#1 and siRNA#2 produced consistent knockdown results in MHCC97H and HepG2 cell lines, so they were selected for follow-up studies. To determine the effect of NAP1L5 on the function of HCC cells, we performed a variety of *in vitro* experiments. EDU staining, colony formation assays, Transwell assays and wound healing migration assays all showed consistent results: the downregulation of NAP1L5 significantly promoted the proliferation, invasion and migration of MHCC97H and HepG2 cells (Figure 2D–2G). We used plasmids to overexpress NAP1L5 in MHCC97H and HepG2 cells. The efficiency of NAP1L5 overexpression was confirmed by comparison with the vector at the mRNA and protein levels (Figure 3A, 3B). Repeating the above experiments, it was found that overexpression of NAP1L5 significantly inhibited the proliferation, invasion and migration of MHCC97H and HepG2 cells (Figure 3C–3F). These results are consistent with the downregulated data of NAP1L5, suggesting that NAP1L5 may be a key factor in the regulation of hepatoma cell function.

### NAP1L5 induces a decrease in S phase of the cell cycle and promotes apoptosis *in vitro*

**Figure 4:**
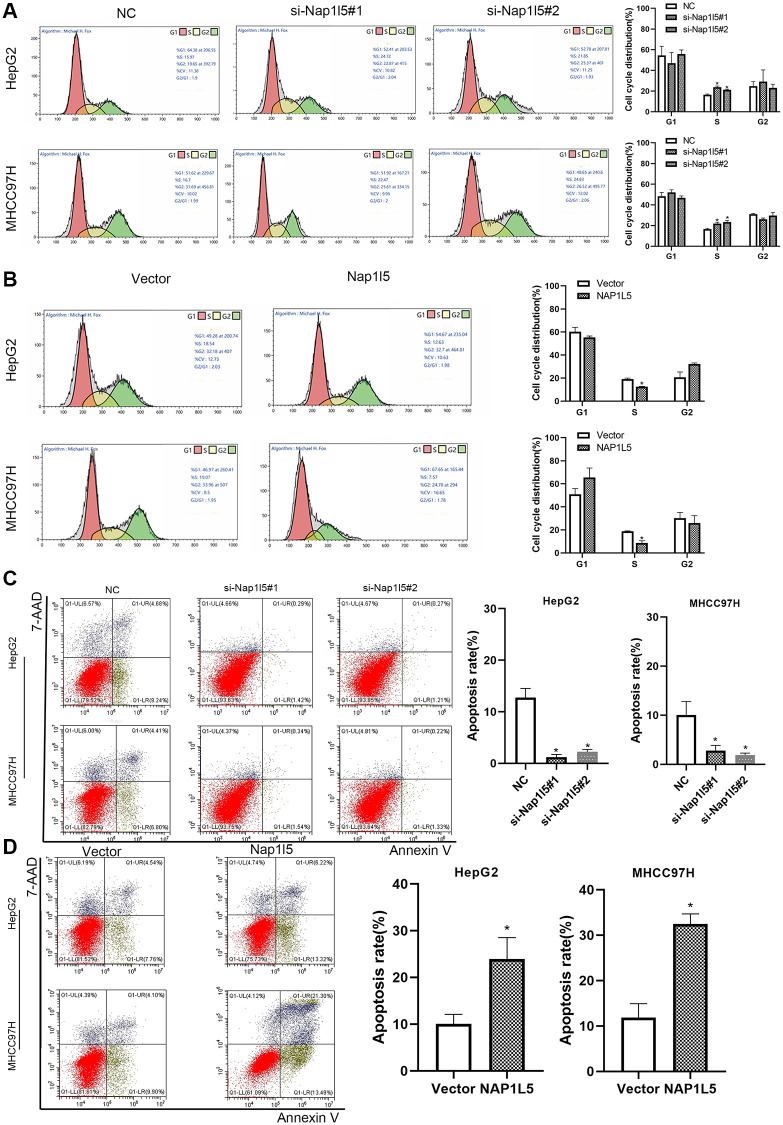
**NAP1L5 induces cell cycle S phase cell reduction and promotes apoptosis *in vitro*.** (**A**) The effect of NAP1L5 downregulation on the cell cycle distribution of HepG2 and MHCC97H cells. (**B**) The effect of NAP1L5 overexpression on the cell cycle distribution of HepG2 and MHCC97H cells. (**C**) The effect of the downregulation of NAP1L5 on the apoptosis of HepG2 and MHCC97H cells. (**D**) The effect of NAP1L5 overexpression on HepG2 and MHCC97H cell apoptosis. ^*^p < 0.05; ^**^p < 0.01; ^***^p < 0.001.

We measured the cell cycle distribution by flow cytometry and found that the percentage of S phase cells downregulated by NAP1L5 among HepG2 and MHCC97H cells was significantly higher than that in the control group (Figure 4A). In contrast, in HepG2 and SMMC7721 cells overexpressing NAP1L5, the number of cells decreased in S phase (Figure 4B). Flow cytometry analysis using Annexin V-APC/7-AAD staining showed that the downregulation of NAP1L5 inhibited apoptosis in the two cell lines (Figure 4C), while the upregulation of NAP15 promoted apoptosis (Figure 4D). The above data suggest that NAP1L5 can induce a decrease in S phase cells and promote the apoptosis of HCC cells.

### NAP1L5 inhibits tumor growth and metastasis *in vivo*

**Figure 5:**
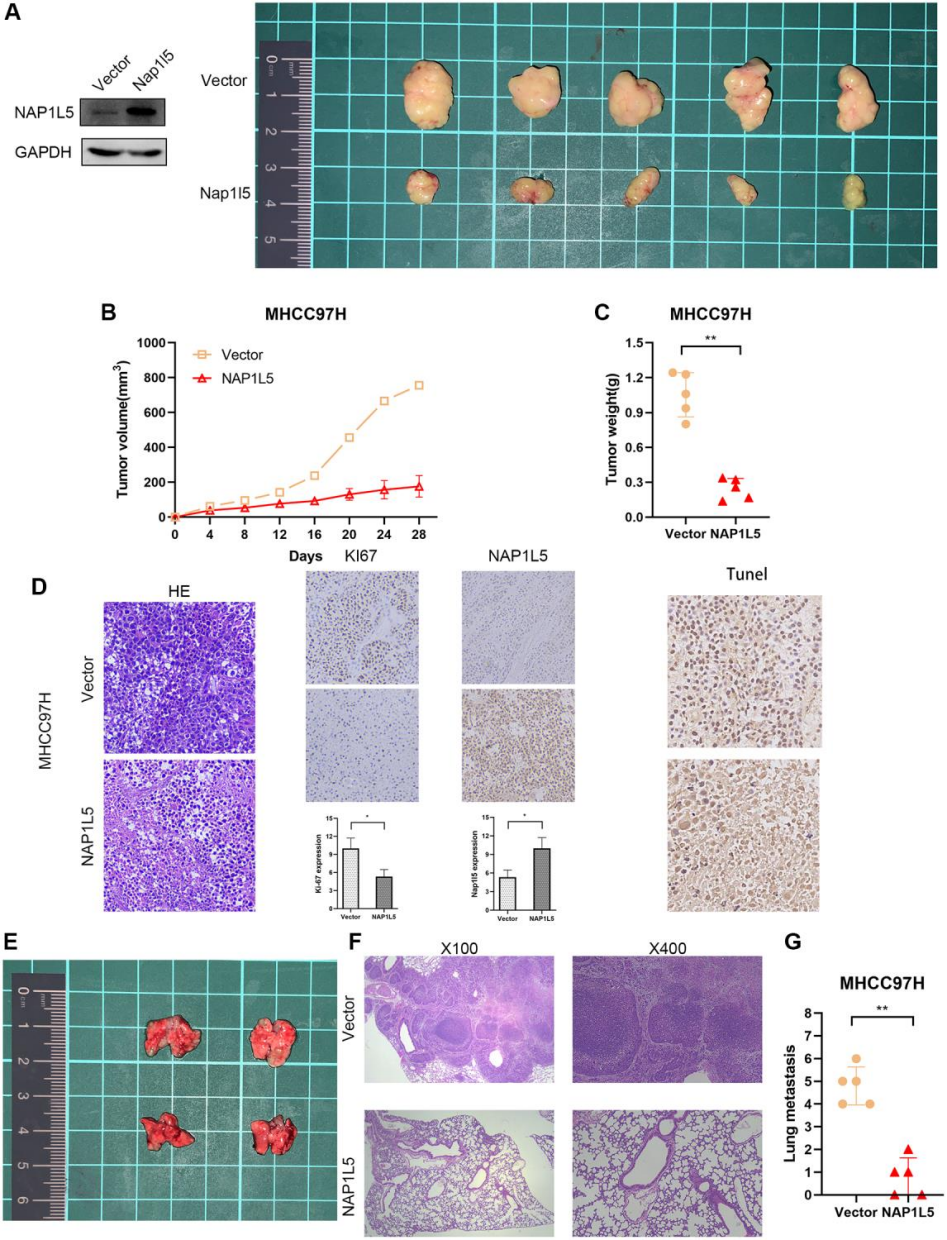
**NAP1L5 inhibits tumor growth and metastasis *in vivo*.** (**A**–**C**) Xenografted tumors were produced by injection of MHCC97H cells overexpressing NAP1L5 (experimental group) or carrying control vector (control group). The growth of the transplanted tumor was measured by volume and weight. (**D**) HE, IHC, Ki-67 and TUNEL staining were performed on the transplanted tumor. (**E**) The lung metastasis model was established by injecting MHCC97H cells overexpressing NAP1L5 (experimental group) or control vector (control group) through the tail vein. (**F**, **G**)The number of metastatic foci in lung tissue was observed, and HE staining was performed on lung tissue. ^*^p < 0.05; ^**^p < 0.01; ^***^p < 0.001.

MHCC97H cells stably overexpressing NAP1L5 were implanted into nude mice in the experimental group, and MHCC97H cells transferred into the control vector were implanted into nude mice in the control group (MHCC97H cells transfected with lentivirus vector were verified by Western blotting), and the proliferation of tumor cells *in vivo* was observed. All mice were monitored every 4 days, and the mice were killed four weeks later. The average tumor weight and volume of mice in the experimental group decreased significantly in the fourth week (Figure 5A–5C). The transplanted tumors of the two groups underwent HE and immunohistochemical staining of Ki-67, NAP1L5 and TUNEL. We found that the Ki67 score was lower in the experimental group, while the NAP1L5 and TUNEL scores were higher (Figure 5D). To explore the effect of NAP1L5 on tumor metastasis, MHCC97H cells carrying the NAP1L5 lentivirus vector or control vector were injected into the experimental and control groups through the tail vein. The mice were killed 4 weeks later, and the lung tissue was taken to observe the number of metastatic foci and stained by HE. We found that the overexpression of NAP1L5 was significantly associated with reduced lung metastasis in mice (Figure 5E). In addition, we performed a pathological analysis of the lung tissue of nude mice, and the results showed that the incidence of lung metastasis in the experimental group was lower than that in the control group (Figure 5F, 5G). The above data further confirmed the antitumor activity of NAP1L5 against hepatocellular carcinoma.

### NAP1L5 inhibits the progression of hepatocellular carcinoma through the PI3K/AKT/MTOR signaling pathway

**Figure 6:**
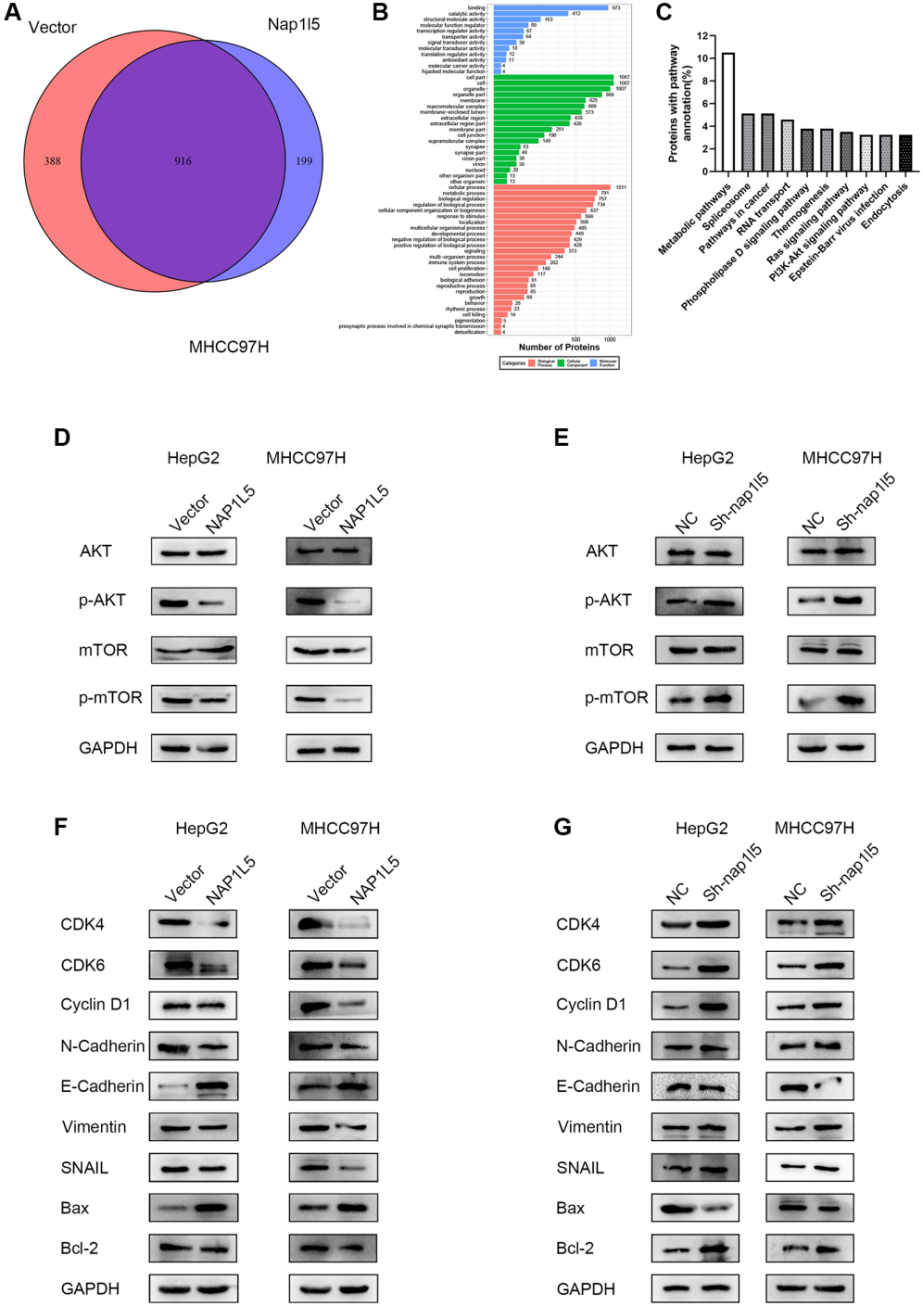
**NAP1L5 regulates the PI3K/AKT/MTOR signaling pathway in hepatoma cells.** (**A**) Venn diagram of the mass spectrometry analysis of MHCC97H cells overexpressing NAP1L5 and transferred into the control vector. (**B**) Mass spectrometry analysis of the GO functional enrichment ma*p* of MHCC97H cells overexpressing NAP1L5. (**C**) Enrichment of differentially expressed gene pathways in NAP1L5-overexpressing and MHCC97H cells transfected with the control vector. (**D**) Western blotting was used to detect the expression of p-AKT and p-mTOR in HepG2 and MHCC97H cells overexpressing NAP1L5. (**E**) Western blotting was used to detect the expression of p-AKT and p-mTOR in HepG2 and MHCC97H cells downregulated by NAP1L5. (**F**) Western blotting was used to analyze the expression of key molecules involved in cell cycle regulation, EMT and apoptosis in HepG2 and MHCC97H cells overexpressing NAP1L5. (**G**) Western blotting was used to analyze the expression of key molecules involved in cell cycle regulation, EMT and apoptosis in HepG2 and MHCC97H cells downregulated by NAP1L5.

The MHCC97H cells carrying the control and lentivirus vectors with high expression of NAP1L5 were enriched with 916 genes by mass spectrometry, while GO enrichment analysis showed that there were 199 independent differentially expressed genes after NAP1L5 overexpression compared with the control vector group (Figure 6A). The GO function analysis of the two groups of cells is shown in Figure 6B. The independent difference in 199 genes was highly correlated with the PI3K/AKT/MTOR signaling pathway (Figure 6C). We determined the key proteins in the PI3K/AKT/MTOR signaling pathway and found that overexpression of NAP1L5 decreased the phosphorylation of AKT and mTOR in HepG2 and MHCC97H cells (Figure 6D), indicating that PI3K/AKT/mTOR signaling was inhibited. In contrast, NAP1L5 knockdown enhanced the PI3K/AKT/mTOR signaling activity (Figure 6E). In addition, the overexpression of NAP1L5 changes the expression of indispensable key regulators in cell cycle regulation, epithelial-mesenchymal transition (EMT) and apoptosis. Western blotting showed that the expression of cyclin D1; CDK4; CDK6; EMT-related proteins N-cadherin, vimentin, and SNAIL; and apoptosis-related protein Bcl-2 decreased in HepG2 and MHCC97H cells overexpressing NAP1L5, while the levels of E-cadherin and Bax were upregulated (Figure 6F). The Western blotting results in the cells downregulated by NAP1L5 were the opposite (Figure 6G).

**Figure 7:**
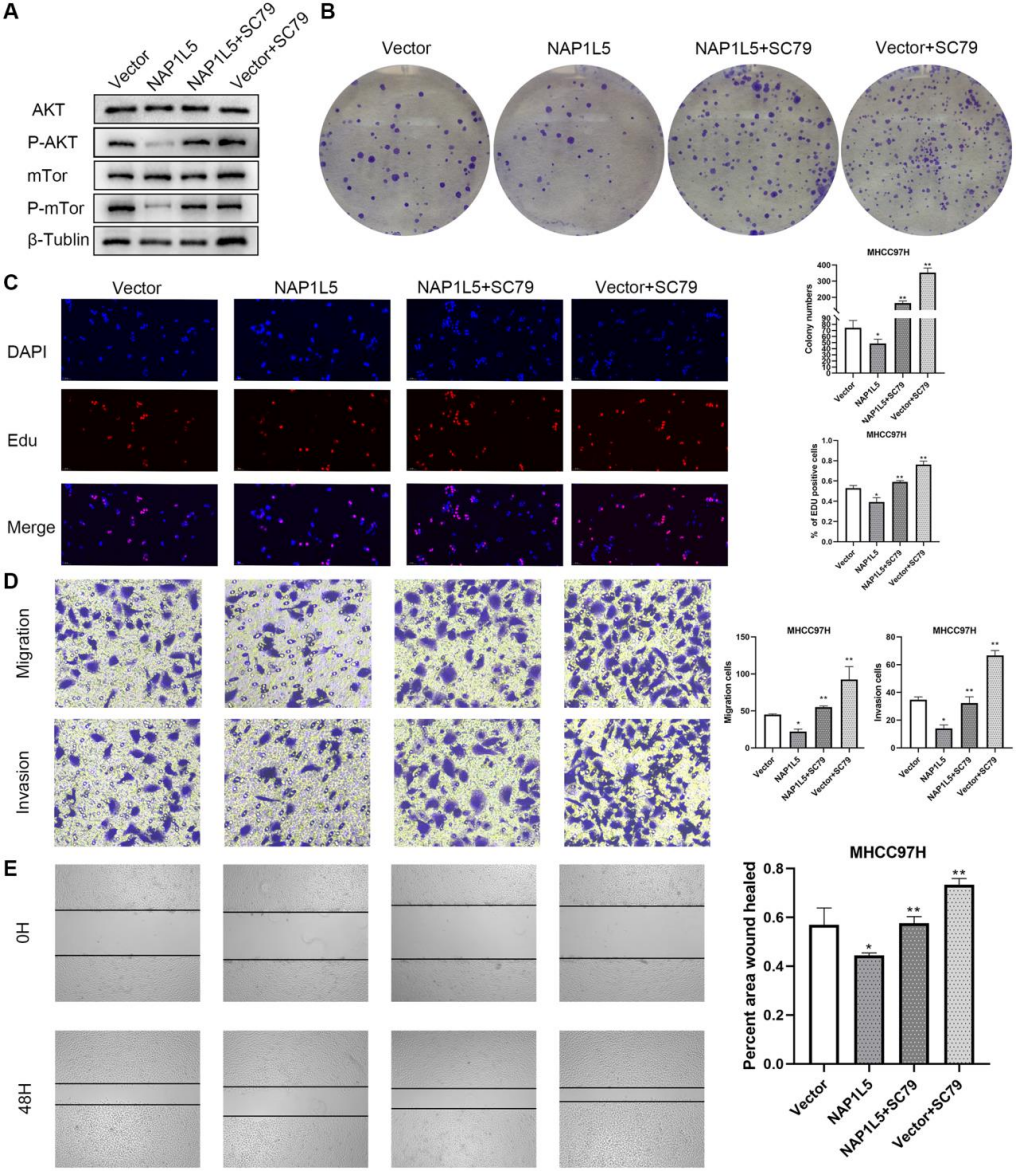
**NAP1L5 inhibits the progression of hepatocellular carcinoma by regulating PI3K/AKT/MTOR.** (**A**) The expression of p-AKT and p-mTOR in MHCC97H-Vector cells and MHCC97H-NAP1L5 cells treated with SC79 (100 ng/mL) and the two types of cells without SC79 treatment was detected by Western blotting. (**B**) A colony formation assay was used to detect the effect of SC79 (100 ng/mL) on the proliferation of MHCC97H-Vector and MHCC97H-NAP1L5 cells. (**C**) EdU staining was used to detect the effect of SC79 (100 ng/mL) on the proliferation of MHCC97H-Vector and MHCC97H-NAP1L5 cells. (**D**) The effect of SC79 (100 ng/mL) on the migration and invasion of MHCC97H-Vector and MHCC97H-NAP1L5 cells was detected by a Transwell assay. (**E**) The effect of SC79 (100 ng/mL) on the migration of MHCC97H-Vector and MHCC97H-NAP1L5 cells was detected by a wound healing migration assay. ^*^p < 0.05; ^**^p < 0.01; ^***^p < 0.001.

We promoted the activity of the PI3K/AKT/mTOR signaling pathway by SC79 treatment, and the reduction in p-AKT and p-mTOR was restored after adding SC79 to NAP1L5-overexpressing MHCC97H cells (Figure 7A). It was also found that SC79 induced and restored the inhibitory effect of NAP1L5 overexpression on the proliferation, migration and invasion of MHCC97H cells (Figure 7B–7E). In summary, these data suggest that NAP1L5 inhibits HCC progression by inhibiting the PI3K/AKT/mTOR signaling pathway.

### NAP1L5 combined with MYH9 inhibits the PI3K/AKT/MTOR axis and HCC progression

**Figure 8:**
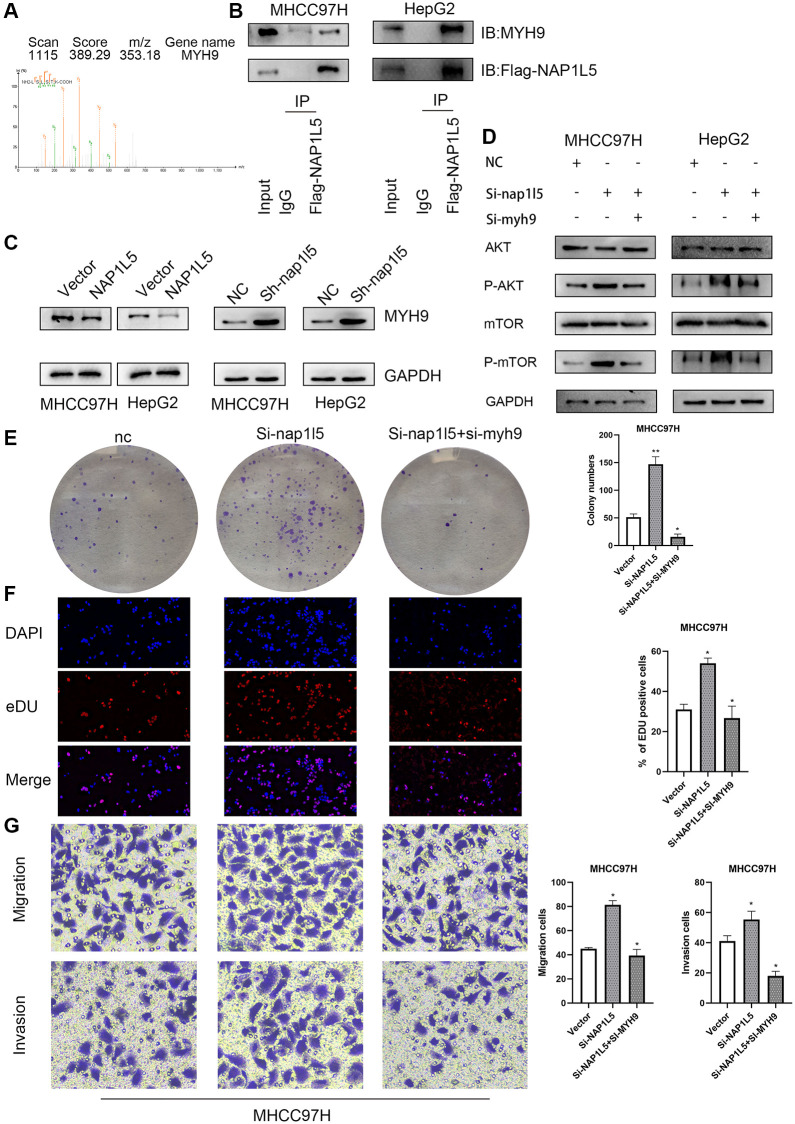
**Effects of NAP1L5 binding to MYH9 on downstream signaling pathways and cell function in hepatocellular carcinoma.** (**A**) The possible interacting proteins were analyzed by mass spectrometry. Trypsin digestion fragments were detected by mass spectrometry, and B and Y represent N-terminal and C-terminal collision-induced dissociation fragment ions. (**B**) Co-IP confirmed the combination of NAP1L5 and MYH9. (**C**) Western blotting showed that the expression of NAP1L5 protein was negatively correlated with that of MYH9 protein. (**D**) Both NAP1L5 and MYH9 were downregulated in the MHCC97H cell line. Western blotting showed that the phosphorylation levels of AKT and mTOR were lower than those in the NAP1L5-downregulated cell line. (**E**) A colony formation assay verified the change in the proliferation ability of MHCC97H cells with simultaneous downregulation of NAP1L5 and MYH9. (**F**) EdU staining verified the change in the proliferation ability of MHCC97H cells with simultaneous downregulation of NAP1L5 and MYH9. (**G**) A Transwell assay verified the change in the migration and invasion ability of MHCC97H cells with simultaneous downregulation of NAP1L5 and MYH9. ^*^p < 0.05; ^**^p < 0.01; ^***^p < 0.001.

Further mass spectrometry analysis revealed that MYH9 was a protein captured by NAP1L5 (Figure 8A). Co-IP verified the specificity of this interaction, indicating that NAP1L5 could bind to MYH9 (Figure 8B). Overexpression of NAP1L5 resulted in a decrease in MYH9 protein levels in MHCC97H and HepG2 cells (Figure 8C). In contrast, NAP1L5 knockdown led to an increase in MYH9 protein (Figure 8C). Many previous studies have confirmed that MYH9 promotes the occurrence and development of hepatocellular carcinoma, such as the research of Professor Fang Weiyi of Southern Medical University on the role of MYH9 in HCC [12, 13]. The promotive role of MYH9 in hepatocellular carcinoma was confirmed, in accordance with previous studies. Knockdown of both NAP1L5 and MYH9 and downregulation of MYH9 genes in MHCC97H cells weakened the ability to promote phosphorylation of AKT and mTOR formed by low expression of NAP1L5 (Figure 8D) and restored the enhanced proliferation, migration and invasion of HCC cells (Figure 8E–8G). These data demonstrate that NAP1L5 inhibits the progression of hepatocellular carcinoma and PI3K/AKT/MTOR signaling via MYH9.

**Figure 9:**
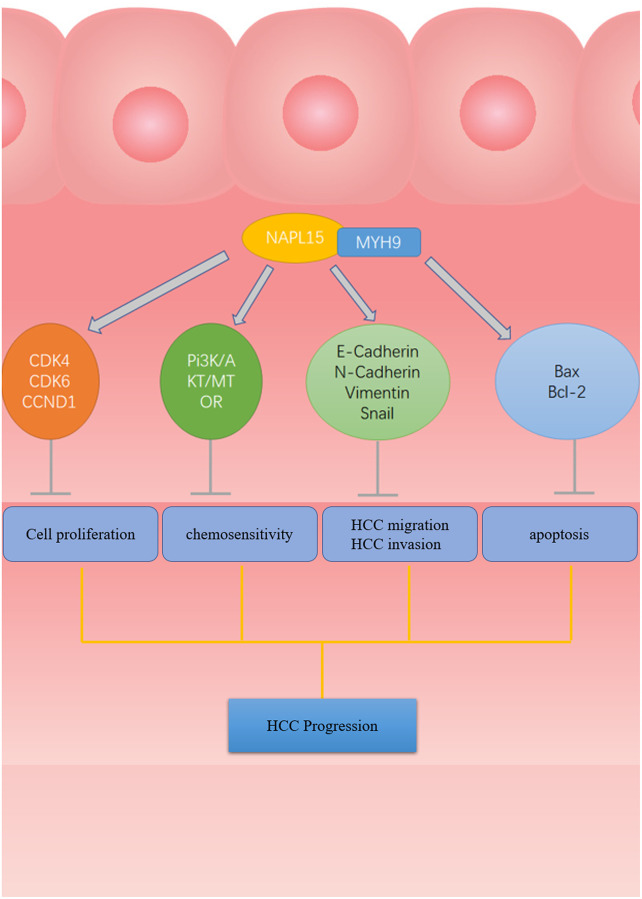
**The flow chart of NAP1L5 targeting combined with MYH9 Inhibit HCC progression.** NAP1L5 targets and binds MYH9 to regulate cycle, apoptosis, EMT-related protein expression and PI3K/AKT/mTOR signal pathway activity, thus inhibiting the progression of hepatocellular carcinoma.

Figure 9 is a schematic diagram that reveals the process by which NAP1L5 binds MYH9 to regulate cycle, apoptosis, EMT-related proteins and downstream PI3K/AKT/mTOR signaling pathways that affect the progression of HCC (Figure 9).

## DISCUSSION

NAP1L5 is the human counterpart of yeast NAP-1 protein, a histone binding factor involved in maintaining the formation of cumulative nucleosomes [14]. Studies have also shown that human NAP1L5 may play a negative role in cell cycle progression, usually inducing cell cycle arrest and inhibiting cell growth [15]. However, there are few studies on NAP1L5. To the best of our knowledge, the relationship between NAP1L5 and HCC has not been reported. In this study, we first proved the downregulation of NAP1L5 in hepatocellular carcinoma tissue and that the downregulation of NAP1L5 is related to shorter overall and disease-free survival times. Then, we confirmed that NAP1L5 significantly inhibited the proliferation, migration and invasion of HCC cells and promoted the apoptosis of HCC cells *in vitro* and *in vivo*. In addition, we found that NAP1L5 may inhibit the progression of hepatocellular carcinoma by inhibiting the PI3K/AKT/MTOR signaling pathway. Finally, we proved that NAP1L5 suppresses PI3K/AKT/MTOR signaling and HCC progression through MYH9 targeting.

To explore the effect of NAP1L5 in HCC, we first detected the expression of NAP1L5 in liver cancer clinical tissue specimens and the public TCGA database. The results showed that the expression of NAP1L5 was downregulated in hepatocellular carcinoma. Through the discussion of the clinicopathological features of the patients, it was found that the expression of NAP1L5 was related to tumor volume, survival time and recurrence (*P* < 0.05), suggesting that NAP1L5 may have antitumor activity in hepatocellular carcinoma. We used plasmids and siRNAs to change the expression of NAP1L5 in HCC cells and verified the effect of this change by EdU staining, colony formation assays, Transwell assays, wound healing migration assays, flow cytometry and animal experiments. The results showed that NAP1L5 could significantly inhibit the proliferation, invasion and metastasis of HCC cells *in vitro* and *in vivo*. In some studies, the overexpression of NAP1L5 significantly promoted the proliferation of 293T cells, while the growth rate of 293T cells downregulated by NAP1L5 decreased. This study suggested that Nap1l5 did not play a negative role in the process of the cell cycle [16]. This is inconsistent with our conclusion, and we speculate that NAP1L5 plays different roles in different tissues and cells.

Through mass spectrometry analysis and GO enrichment analysis, we found that the overexpression of NAP1L5 was highly related to the PI3K/AKT/MTOR signaling pathway and proved that NAP1L5 inhibited the progression of liver cancer through the PI3K/AKT/MTOR signaling pathway. The PI3K/AKT/mTOR signaling pathway has a wide range of functions in HCC and may have wide application prospects in the treatment of liver cancer in the future [17]. The PI3K/Akt/mTOR signaling pathway is overexpressed in nearly 50% of hepatocellular carcinoma cases, and its abnormal activation affects cell proliferation, metabolism, tumor cell differentiation, lipid metabolism, autophagy and EMT [18, 19]. It has been found that the activation of PI3K and Akt leads to the phosphorylation of the transcription factor cyclic adenosine monophosphate (CAMP) response element binding protein (CREB) on Ser133, leading to CREB dimerization and activation, which regulates cell proliferation, apoptosis, angiogenesis, metastasis and metabolism [20]. Foxo1 is the main FOXO protein that promotes the occurrence and development of hepatocellular carcinoma [21]. In hepatocellular carcinoma, the activation of Akt inhibits the transcriptional activity of FOXO1, while FOXO1 usually inhibits the expression of epithelial mesenchymal transformation-induced transcription factor and transforming growth factor-β, leading to epithelial mesenchymal transformation and promoting the migration and invasion of hepatocellular carcinoma cells [22]. The PI3K/AKT/mTOR signaling pathway regulates the NF-κB family of transcription factors, which regulate inflammation, cellular stress, and innate and acquired immune responses, which in turn regulate the survival, proliferation, migration and invasion of hepatocytes, Kupffer cells and hepatic stellate cells [23]. The PI3K/Akt/mTOR signaling pathway can promote lipid synthesis by regulating the transcription factor sterol regulatory element binding protein-1 (SREBP-1) and other mechanisms to support cell growth, proliferation and tumorigenesis [24]. The PI3k/Akt/mTOR signaling pathway also regulates autophagy. Under normal physiological conditions, when mTORC1 is activated, autophagy is inhibited by activating the TG1 human homologs UNC-51-like autophagy activating protein 1 (ULK1) and UNC-51-like autophagy activating protein 2 (ULK2) [25, 26]. This evidence suggests that the activation of the PI3K/Akt/mTOR signaling pathway is involved in the progression of hepatocellular carcinoma. The cellular mechanism of widespread activation of the PI3K/Akt/mTOR pathway in hepatocellular carcinoma is not completely clear, but inhibition of PI3K/AKT/mTOR can prevent abnormal cell proliferation, cell metabolism and tumor angiogenesis, thus providing potential molecular targeted therapy [27]. Inhibitors of this pathway have been the focus of research in the treatment of hepatocellular carcinoma. Studies have confirmed that sorafenib can prolong the median survival time of patients with advanced hepatocellular carcinoma and become a first-line treatment for advanced HCC [28]. The efficacy and safety of lenvatinib are similar to those of sorafenib, and it has become the second first-line systematic treatment for advanced liver cancer [29]. Rapamycin inhibits the phosphorylation of mTOR *in vitro* and *in vivo*, which effectively inhibits the growth and proliferation of hepatocellular carcinoma cells [30]. Currently, it is believed that the crosstalk between the PI3K/AKT/mTORm signaling pathway and other signaling pathways and the existence of a mTOR feedback loop may be the reasons for resistance to treatment [31]. Combined with our research, the above conclusions establish an indirect relationship between NAP1L5 and the downstream variety effects of the PI3K/AKT/mTOR signaling pathways for further research and exploration.

We proved the interaction between NAP1L5 and MYH9 by mass spectrometry and Co-IP and found that they regulated the downstream signaling pathways and functions of hepatocellular carcinoma cells. The MYH9 gene encodes a traditional nonmuscle myosin (NMIIA), which participates in cytoskeleton reorganization, focus contact formation and lipid contraction and regulates cell adhesion and migration [32]. Defects in this gene can cause many diseases, such as MYH9-related diseases (MYH9-RD), chronic kidney disease, and sensorineural deafness [33]. However, tumor research has confirmed that MYH9 can play a dual role in cancer. Studies have shown that in head and neck squamous cell carcinoma, low expression of MYH9 leads to poor prognosis, thus supporting the role of MYH9 as a human tumor suppressor [34]. Other studies have shown that MYH9 binds to CXCR4 and promotes the migration and invasion of renal cell carcinoma [35]. In addition, MYH9 plays a promoting role in the invasion and metastasis of gastric cancer [36, 37] and colorectal cancer [38]. Liver cancer-related data show that Myh9 may promote the dryness of liver cancer and accelerate cancer progression through Wnt signaling [13]. We also reached a consistent conclusion in our study that MYH9 plays a promoting role in liver cancer.

In summary, our research shows that NAP1L5 is expressed at low levels in HCC and that its expression is related to the survival rate of patients, further proving that NAP1L5 regulates MYH9 to inhibit the progression of HCC through the PI3K/AKT/mTOR signaling pathway. This suggests that NAP1L5 may be a new biomarker with which to evaluate the prognosis of liver cancer and a potential therapeutic target for liver cancer.
